# Clinical Protocols for the Initial Evaluation and Follow-Up of Patients with Chronic Chagas Disease: A Proposal for Referral Centers

**DOI:** 10.3390/tropicalmed11010003

**Published:** 2025-12-20

**Authors:** Alejandro Marcel Hasslocher-Moreno, Ana Cristina Ribeiro Rohem, Andrea Rodrigues da Costa, Andréa Silvestre de Sousa, Fernanda de Souza Nogueira Sardinha Mendes, Fernanda Martins Carneiro, Flavia Mazzoli-Rocha, Gilberto Marcelo Sperandio da Silva, Henrique Horta Veloso, Luciana Fernandes Portela, Luiz Henrique Conde Sangenis, Marcelo Teixeira de Holanda, Paula Simplicio da Silva, Roberto Magalhães Saraiva, Sergio Salles Xavier, Mauro Felippe Felix Mediano

**Affiliations:** Evandro Chagas National Institute of Infectious Diseases, Oswaldo Cruz Foundation, Rio de Janeiro 21040-360, Brazil; alejandro.hasslocher@ini.fiocruz.br (A.M.H.-M.); ana.rohem@ini.fiocruz.br (A.C.R.R.); andrea.costa@ini.fiocruz.br (A.R.d.C.); andrea.silvestre@ini.fiocruz.br (A.S.d.S.); fernanda.sardinha@ini.fiocruz.br (F.d.S.N.S.M.); fernanda.martins@ini.fiocruz.br (F.M.C.); flavia.mazzoli@ini.fiocruz.br (F.M.-R.); gilberto.silva@ini.fiocruz.br (G.M.S.d.S.); henrique.veloso@ini.fiocruz.br (H.H.V.); luciana.portela@ini.fiocruz.br (L.F.P.); luiz.sangenis@ini.fiocruz.br (L.H.C.S.); marcelo.holanda@ini.fiocruz.br (M.T.d.H.); paula.simplicio@ini.fiocruz.br (P.S.d.S.); roberto.saraiva@ini.fiocruz.br (R.M.S.); sergio.xavier@ini.fiocruz.br (S.S.X.)

**Keywords:** chagas disease, clinical evaluation, therapeutic pathway, clinical protocol, comprehensive health care

## Abstract

Chagas disease (CD) remains a major global health challenge and requires standardized, multidisciplinary, and evidence-based clinical approaches. This article aims to present and systematize the model of clinical routines developed at the Clinical Research Laboratory on Chagas Disease (Lapclin-Chagas), INI/Fiocruz, for the initial evaluation and longitudinal follow-up of patients with chronic CD. The proposal is intended to serve as a replicable and adaptable framework for referral centers in both endemic and non-endemic settings. Using a descriptive qualitative design, institutional protocols, national and international guidelines, and expert consultations were analyzed to construct a comprehensive care model. The resulting protocol integrates diagnostic pathways (including dual serological confirmation and clinical staging), criteria for etiological treatment, and coordinated multidisciplinary follow-up involving cardiology, gastroenterology, pharmaceutical care, nutrition, psychology, and social support. Specific pathways are also presented for *Trypanosoma cruzi* (*T. cruzi*)/HIV coinfection, laboratory accidents, and monitoring of adverse reactions to benznidazole. By consolidating more than three decades of institutional experience into operational workflows, this proposal offers an innovative contribution to the organization of CD care and provides actionable guidance for health systems seeking to improve diagnostic accuracy, therapeutic adherence, patient safety, and long-term outcomes.

## 1. Introduction

The Clinical Research Laboratory on Chagas Disease (Lapclin-Chagas) of the Evandro Chagas National Institute of Infectious Diseases (INI), Oswaldo Cruz Foundation (Fiocruz), has the mission “to provide high-quality solutions for the care of individuals with Chagas disease, in line with their biopsychosocial context, within the levels of complexity of medical care and the framework of the Brazilian Unified Health System (SUS)”. Its vision is “to be nationally and internationally recognized as a center of excellence in clinical research and in the care of people with Chagas disease”. Consistent with the mission of INI/Fiocruz, Lapclin-Chagas pursues the integrated goals of patient care, research, and teaching. Since 1986, it has served as a referral center for treatment and research on Chagas disease (CD), developing a care model based on the principles of SUS. This model emphasizes patient reception and humanization of care; access to complex, high-quality diagnostic tests and procedures; pharmaceutical services; training and qualification of health professionals; dissemination of information; active patient participation; and community outreach initiatives.

The World Health Organization (WHO) classifies CD, caused by the protozoan *Trypanosoma cruzi* (*T. cruzi*), among the 21 neglected tropical diseases [1]. Endemic regions have traditionally been defined by the distribution of insect vectors transmitting the parasite, spanning 21 continental countries in the Americas. In 2010, the Pan American Health Organization (PAHO) estimated that between 6 and 8 million people were infected with *T. cruzi* in the region, with about 30,000 new vector-borne cases and 8000 congenital cases per year, and approximately 65 million people remained at risk, with an estimated 12,000 deaths annually [2]. In 2025, the WHO estimates that more than 7 million people worldwide are infected with *T. cruzi*, leading to over 10,000 deaths every year, and more than 100 million people are considered at risk of infection [1]. A 2024 update estimated 3.7 million cases in Brazil, predominantly among women [3]. Initially confined to Latin America, CD has now expanded to the around the globe, representing an emerging public health issue in the United States and Europe [4].

Diagnosis of CD integrates epidemiological history, clinical findings, and laboratory testing, depending on the stage of infection. In the acute phase, suspicion arises in individuals with persistent fever (>7 days) and exposure to triatomines or potentially contaminated food. It should also be considered after community outbreaks of oral transmission or in asymptomatic children under three years born to seropositive mothers [5,6,7]. In the chronic phase, suspicion is supported by current or past residence in endemic areas, exposure to infested dwellings, previous history of blood transfusion, family history of CD, or consumption of in natura foods (notably in the Amazon) or game meat [5,8]. Laboratory confirmation in the acute phase relies on direct parasitological detection, while in the chronic phase, the gold standard method is positive *T. cruzi* IgG serology confirmed by two tests with a different methodology [9].

CD progresses through two phases: an acute phase, shortly after infection, and a chronic phase, which persists lifelong if no etiological treatment is provided. Acute manifestations range from nonspecific symptoms to more specific signs, with possible severe complications. Chronic CD is classified into indeterminate (IF), cardiac (CF), and digestive (DF) forms, with a mixed form (MF) when CF and DF coexist [5].

Chronic CD contributes substantially to morbidity and mortality through cardiac and gastrointestinal complications [10]. The chronic phase is dynamic, with an estimated 1.9% annual progression from IF to determinate forms [11] and further progression within CF or DF [12]. PAHO and Brazilian Ministry of Health guidelines recommend mandatory treatment in acute cases and in chronic reactivation among immunosuppressed individuals. In chronic disease, trypanocidal treatment is strongly advised for children, adolescents, and women of childbearing age, as well as adults up to 50 years with preserved myocardial function [9,13]. For older adults, treatment decisions should consider disease duration, absence of severe cardiomyopathy, and patient preferences [9]. Evidence indicates that trypanocidal drugs can alter the natural history of CD, reducing disease progression [14].

Comprehensive health care, integrality, interdisciplinarity, intersectorality, and continuity of care, are a central concept in SUS [15]. This approach emphasizes addressing real health needs effectively rather than “doing everything for everyone”. In CD care, standardized collection of epidemiological data, diagnostic testing, clinical management, and treatment indication are essential for both urban referral centers and rural primary care settings. Such standardization improves diagnostic and therapeutic decisions [16,17].

Chagas disease increasingly represents a challenge for health systems in both endemic and non-endemic countries. Although national and international guidelines provide essential recommendations, few publications offer operational, step-by-step clinical pathways that consolidate diagnostic routines, therapeutic decision-making, and multidisciplinary follow-up into a unified framework that can be adapted by diverse institutions.

The innovative contribution of this work lies in operationalization of care into clear diagnostic and therapeutic workflows illustrated through structured pathways; integration of multidisciplinary teams, including cardiology, gastroenterology, rehabilitation, pharmaceutical care, nutrition, psychology, and social work services, into a coordinated model tailored to the diverse clinical forms of CD; standardization of etiological treatment monitoring, including adverse reaction management and follow-up schedules; specialized pathways for *T. cruzi*/HIV coinfection, accidental exposure, and management of high-risk scenarios; practical applicability for health systems seeking to implement or strengthen CD care, especially in settings lacking established routines. By consolidating long-standing clinical practices into a structured and adaptable protocol, this manuscript aims to support institutions worldwide in improving diagnostic accuracy, treatment adherence, patient safety, and overall quality of care for individuals living with chronic CD.

The main objective of this manuscript is to present, systematize, and validate the clinical routines developed at INI/Fiocruz for the initial evaluation and longitudinal follow-up of patients with chronic CD, offering a model that can be replicated in referral centers of varying levels of complexity around the world. This proposal synthesizes institutional experience accumulated since 1986 and integrates updated scientific evidence, guideline recommendations, and expert knowledge into a cohesive protocol.

## 2. Materials and Methods

This study is a descriptive, qualitative investigation aimed at systematizing the clinical routines adopted for the initial evaluation and follow-up of patients with chronic CD at INI/Fiocruz. The formulation of these routines followed a structured five-stage methodology:

### 2.1. Document Review

Two key institutional documents were reviewed: the “Activity Report of the National Institute of Infectious Diseases Evandro Chagas, 2013–2017” (available at https://www.researchgate.net/publication/370581072, accessed on 12 October 2025) and the “Institutional Strategic Plan of the National Institute of Infectious Diseases Evandro Chagas”, updated in 2023 (available at https://www.ini.fiocruz.br/, accessed on 12 October 2025). These documents contain information on the main guidelines related to healthcare delivery, clinical research, and educational activities, both general and specific to each Clinical Research Laboratory at INI/Fiocruz. Additionally, clinical evolution data extracted from electronic medical records, operational logs documenting outpatient care workflows, and internal manuals used at INI/Fiocruz, covering the period from 1990 to 2025, were accessed and analyzed. This comprehensive review enabled the identification of the examinations, procedures, and care pathways most frequently employed in the triage and clinical evaluation of patients with chronic Chagas disease.

### 2.2. Review of Guidelines and Scientific Literature

National recommendations for the clinical management of CD, including Brazilian Ministry of Health documents and national consensus statements, were consulted to align institutional practices with current evidence and standards of care [5,18,19].

### 2.3. Specialist Consultation

These experts contributed specialized clinical and operational insights to validate routines, refine care pathways, and ensure that the Strategic Démarche accurately incorporated the practical requirements and accumulated experience of each area. Each specialist provided domain-specific expertise: cardiologists offered guidance on diagnostic technologies and the management of Chagas cardiomyopathy; infectious disease specialists informed the overarching clinical approach and etiological treatment; gastroenterologists contributed to the assessment and care of digestive involvement; pharmacists supported medication-use processes and pharmaceutical care routines; social workers identified socioeconomic and access barriers affecting adherence; psychologists addressed psychosocial needs and the mental-health implications of chronic disease; and nutritionists provided input on dietary management and nutritional risks.

### 2.4. Modeling and Internal Validation

The collected information was used to design a structured clinical protocol, organized by stages of care. This proposal underwent review and validation by INI/Fiocruz technical coordination through collective discussion and institutional approval.

### 2.5. Continuous Update

The clinical routines presented are considered dynamic documents and were subject to continuous revisions considering new scientific evidence, emerging diagnostic technologies, and updated public health guidelines.

## 3. Results

The results encompass a set of structured patient care pathways for chronic CD at INI/Fiocruz, covering diagnostic evaluation, initial outpatient management, and follow-up tailored to the clinical form of the disease. Additional flows address etiological treatment with benznidazole (BZN), *T. cruzi*/HIV coinfection, and accidental exposure. Complementary pathways were also defined for cardiopulmonary rehabilitation, pharmaceutical care, nutritional counseling, psychological assessment, and social work support, ensuring an integrated and multidisciplinary approach.

### 3.1. Patient Management for the Diagnostic Assessment of Chagas Disease

Adult patients referred for diagnostic evaluation of CD are first received at the initial care unit of the INI/Fiocruz outpatient clinic, where they are registered in the electronic medical record system. At this stage, two serological tests, using distinct methods, for CD and an electrocardiogram (ECG) are requested, and a follow-up appointment is scheduled within 15 days at the CD outpatient clinic. During this visit, patients undergo disease-specific anamnesis, physical examination, and review of test results, which are then communicated to patients. Patients are also instructed to bring any prior exams related to the suspected diagnosis of CD (ECG, echocardiogram [ECHO], endoscopy, colonoscopy, and/or contrast radiographs of the esophagus and colon), as well as the medical referral that justified the request, when applicable. Importantly, even when a prior diagnosis of CD has been established at another institution, patients must follow this initial care pathway at the INI/Fiocruz outpatient clinic.

Indications for chronic CD investigation encompass both clinical and epidemiological criteria. Cardiological and digestive symptoms, compatible radiological, electrocardiographic, and echocardiographic findings, positive serological tests for *T. cruzi* in blood banks, and a history of having lived in rural or endemic areas are among the most common indicators for suspecting chronic CD. The main indications are summarized in Box 1.

Box 1 **Indications for diagnostic evaluation of chronic Chagas disease.**

History of documented acute CD.

Household contacts, especially mothers or siblings with CD.

History of living in a dwelling with conditions favorable to vector presence (adobe, wattle and daub, thatch, or wooden structures).

Adult patients with conduction disorders and/or arrhythmias (with or without symptoms), or segmental/diffuse myocardial alterations, or heart failure, and with epidemiological history compatible with CD.

Clinical or radiological evidence of acquired esophageal motility disorder or achalasia.

Signs of acquired colonic motility disorders or anatomical deformities.

Reactive serological screening for CD in blood donation.

History of blood transfusion, especially if performed before 1992 (year in which blood blank control was established in Brazil).

Organ transplantation, either as recipient or donor.

For the diagnosis of CD, serological testing includes ELISA *T. cruzi* IgG using total or crude antigen (parasite lysate) and indirect chemiluminescence with recombinant proteins (Abbott ARCHITECT). All assays are performed at the Immunodiagnostic Unit of INI/Fiocruz. For diagnostic confirmation, both methodologies applied simultaneously to the same sample must yield reactive (positive) results. In cases of discordant or indeterminate results, a second sample is analyzed with the same methods [20]. Persistently inconclusive results after two samples warrant a third collection, which is referred to the Laboratory of Parasitic Diseases at the Oswaldo Cruz Institute (IOC-Fiocruz) for indirect immunofluorescence testing.

### 3.2. Initial Patient Management in the Chagas Disease Outpatient Clinic

Patients with positive serology for CD receive counseling on the condition and are invited to begin follow-up and treatment at INI/Fiocruz. A record is created in the electronic medical system.

Baseline tests are requested, including complete blood count with blood typing, biochemistry, liver function panel, lipid profile, thyroid-stimulating hormone (TSH) and free thyroxine (T4), urine test and stool parasitology.

An ECG and an ECHO are requested. In addition, a 24 h Holter monitor is indicated for patients presenting with palpitations or low-output symptoms, and frequent ventricular or supraventricular arrhythmias, or second- to third-degree atrioventricular conduction disorders detected on ECG.

A follow-up visit is scheduled within 30 days. Patients without evidence of cardiac involvement are managed by an infectious disease specialist, whereas those diagnosed with the CF are referred to a cardiologist.

**Figure 1:**
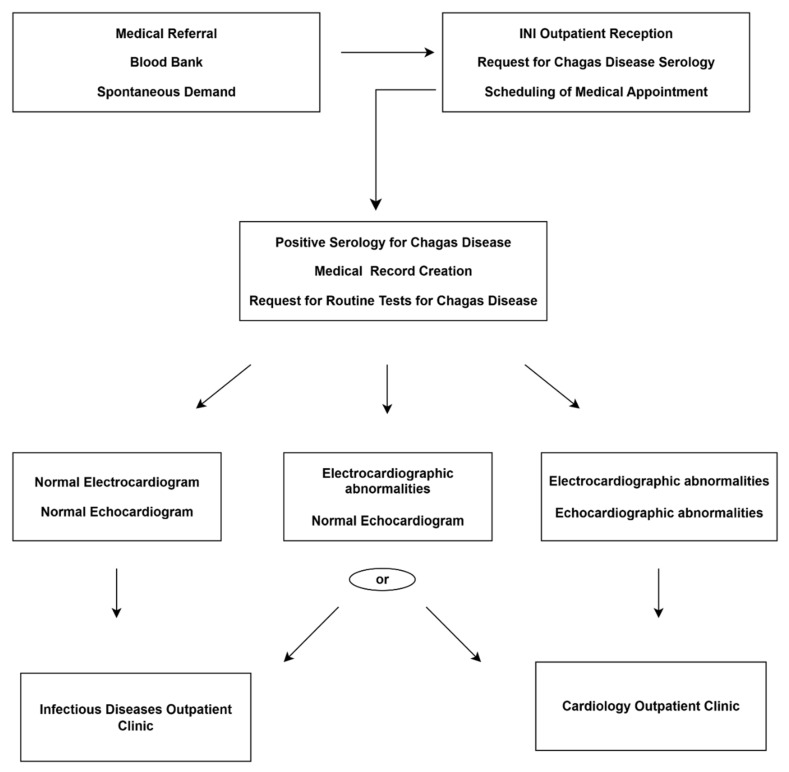
Diagnostic evaluation and initiation of patient follow-up.

Patients with CF are further stratified by the cardiologist according to cardiomyopathy stages (A, B1, B2, C, and D) [5]. Patients identified with DF are referred to a gastroenterologist and, when surgical management of megaesophagus or megacolon is required, they are subsequently directed to general surgery or coloproctology services. (Figure 1).

All patients are evaluated for eligibility for trypanocidal therapy in accordance with current guidelines [9].

Patients with comorbidities are referred to appropriate specialists.

Those with diabetes mellitus, dyslipidemia, metabolic syndrome, or obesity are directed to the nutrition service for continuous monitoring and dietary counseling.

In addition, all patients are referred to social work to facilitate access potential benefits and support programs.

### 3.3. Patient Follow-Up in the Chagas Disease Outpatient Clinic

Once enrolled in long-term care at Lapclin-Chagas, patients remain under continuous follow-up, with no defined time for discharge. The follow-up protocol is tailored to the clinical form of CD. Clinical events arising between scheduled visits are managed at INI/Fiocruz through its urgent and emergency care unit. When hospitalization is necessary, patients are admitted to the INI/Fiocruz hospital center, which provides general wards and an intensive care unit to ensure continuity and support of the outpatient program.

#### 3.3.1. Indeterminate Form

Patients with the IF of CD usually have a favorable prognosis as long as the ECG remains normal. Nonetheless, a proportion may progress to the CF over time. To monitor disease progression, annual ECGs are recommended, and when feasible, a baseline ECHO should be obtained and repeated every five years, or according to physician’s judgment. Given the continuous aging of this population over the last decades, comorbidities are frequent and should be managed according to clinical guidelines. In the absence of comorbidities, follow-up is conducted annually. Etiological treatment with BZN is recommended for this group, with particular emphasis on patients younger than 50 years [9].

#### 3.3.2. Cardiac Form

Following the initial evaluation, patients with findings consistent with CF, also known as chronic Chagas cardiomyopathy (CCC), require staging cardiac involvement to guide diagnostic evaluation, pharmacological management, and prognosis, with particular focus on risk stratification for cardiovascular events and mortality [18]. Patient counseling and education are also essential [21].

Baseline evaluation includes a comprehensive clinical assessment for cardiac symptoms, together with epidemiological and social history [22]. General laboratory is performed to identify comorbidities such as hypertension, diabetes mellitus, dyslipidemia, obesity, renal impairment, and thyroid disorders [23]. Measurement of natriuretic peptides (BNP or NT-proBNP) is recommended in suspected cases of heart failure (HF), both for diagnostic and prognostic purposes [24].

Additional tests at the first visit include chest radiography with contrast esophagus and ECHO. The ECHO is mandatory in the presence of ECG abnormalities, and 24 h Holter monitoring is indicated for evaluation of brady or tachyarrhythmias. Invasive electrophysiological study may be indicated to clarify 24 h Holter-detected abnormalities (e.g., sinus node dysfunction, AV block, alternating bundle branch block, or complex ventricular arrhythmias) or unexplained arrhythmic symptoms such as palpitations, or low-output symptoms such as syncope.

Patients with suspected coronary artery disease (CAD) require appropriate diagnostic testing in accordance with guidelines. Exercise testing has limited diagnostic accuracy for detecting CAD in CCC, as chronic fibrosing myocarditis can cause repolarization abnormalities and areas of electrical inactivity that mimic ischemic heart disease. It is more valuable for evaluating functional capacity and identifying exercise-induced arrhythmias as well as for assessing maximal oxygen consumption, which helps determine eligibility for cardiac transplantation in patients with heart failure. Myocardial scintigraphy is not recommended, as fibrosis patterns may mimic CD-related changes. For patients with high pretest probability of CAD, invasive (angiography) or noninvasive (CT angiography) anatomical assessment is preferred [25]. Cardiopulmonary exercise testing is routinely indicated in patients with HF due to CCC (stage C) and in those referred for cardiac rehabilitation with left ventricular (LV) systolic dysfunction.

All patients with the CF should undergo annual ECG. The ECHO is pivotal for staging, identifying complications, assessing prognosis, and monitoring treatment response [26]. ECHO detects diffuse or segmental wall motion abnormalities (including LV aneurysms), evaluates LV systolic and diastolic function, right ventricular performance, and atrioventricular regurgitation LV ejection fraction is the most important predictor of cardiovascular outcomes and provides prognostic information for all-cause mortality, sudden death, stroke, and HF. ECHO can identify patients at increased risk of atrial fibrillation, detect intracardiac thrombi and monitor their resolution with anticoagulation, as well as demonstrate reverse remodeling following optimized heart failure therapy. Serial ECHOs are therefore essential to guide interventions that may modify the natural history of CCC.

Echocardiography frequency varies by stage: every two years in stage A (normal ECHO); every two years in stage B1 (abnormal ECHO with LVEF >45%); and annually in stage B2 (abnormal ECHO with LVEF ≤45%), as well as in stages C and D. Echocardiography should also be repeated whenever new ECG changes, cardiovascular events (e.g., pacemaker implantation), treatment optimization, or new symptoms occur.

Follow-up intervals are stage-specific: every 6 months in stage A, every 4 months in stage B1, every 3 months in stages B2 and as clinically indicated in stages C and D. Additional visits may be required in cases of cardiovascular complications, anticoagulation management, or presence of comorbidities.

##### Cardiac Exams

All cardiac exams at INI/Fiocruz are requested during medical consultations using the electronic record system, which integrates exam requests, scheduling, and follow-up. ECGs are performed on weekdays using digital equipment by nursing staff trained according to standardized protocols. ECG traces are uploaded to the system for review and formal interpretation by Lapclin-Chagas cardiologists, with validated reports incorporated into the patient’s medical record.

In addition, nursing staff provide orientation before and after all procedures related to cardiac exams, manage the placement and removal of 24 h Holter and ambulatory blood pressure monitoring devices. They are also responsible for assisting in the preparation, monitoring, and recovery of patients undergoing exercise testing and cardiopulmonary exercise testing, with patient safety prioritized throughout the process.

ECHOs are carried out exclusively by Lapclin-Chagas cardiologists, all certified as cardiology specialists and accredited in echocardiography. This structure ensures high-quality diagnostic evaluation, standardization of procedures, and integration of results, contributing to comprehensive care, timely decision-making, and appropriate clinical management of patients.

#### 3.3.3. Digestive Form

Digestive involvement in CD affects 15–30% of patients and is marked by motility disorders leading to akinesia and progressive dilation of gastrointestinal hollow organs, most commonly manifesting as megaesophagus or megacolon. Less frequently, the stomach may be affected, resulting in gastroparesis. Patients presenting with dysphagia are evaluated with barium esophagography and/or upper endoscopy, while those with chronic constipation persisting for more than five days undergo barium enema and/or colonoscopy. Management of DF may be clinical, with dietary adjustments, laxatives, or endoscopic dilations, or surgical in advanced stages. Early detection of digestive “mega” forms is critical to preventing severe complications [12].

### 3.4. Etiological Treatment

In Brazil, BZN is the drug of choice for etiological treatment of patients with the IF of CD and those with the CF in stages A and B1. Etiological treatment for CD is provided free of charge by the Brazilian Ministry of Health to all eligible patients, following notification of the case (acute, chronic, or reactivated) in the Ministry’s online information system. BZN is provided by Laboratório Farmacêutico do Estado de Pernambuco (LAFEPE) and supplied as 100 mg oral tablets, administered in two or three daily doses. In adults, the standard treatment regimen is 5 mg/kg/day for 60 days. A fixed dose of 300 mg per day may be maintained, and the treatment duration can be extended to 80 days depending on body weight [9]. Another drug that can be used when benznidazole is contraindicated or not tolerated is nifurtimox, available in 30 mg and 120 mg tablets and administered orally in two to three daily doses. The recommended dosage is 8–10 mg/kg/day for adults and 10–20 mg/kg/day for children, with a treatment duration of 60 days [9].

At INI/Fiocruz, benznidazole has been the only trypanocidal drug employed. Nifurtimox, in contrast, has never been used in our service.

The most frequent adverse drug reactions (ADRs) associated with BZN include dermatological reactions, mainly allergic dermatitis; neurological effects such as peripheral neuropathy; gastrointestinal symptoms including anorexia, nausea, and vomiting; hepatic abnormalities such as elevated transaminases; and hematological alterations like leukopenia. Gastrointestinal manifestations typically occur within the first week of therapy, dermatitis between the first and second week, and neuropathy usually occurs during the later weeks of treatment [27].

**Figure 2:**
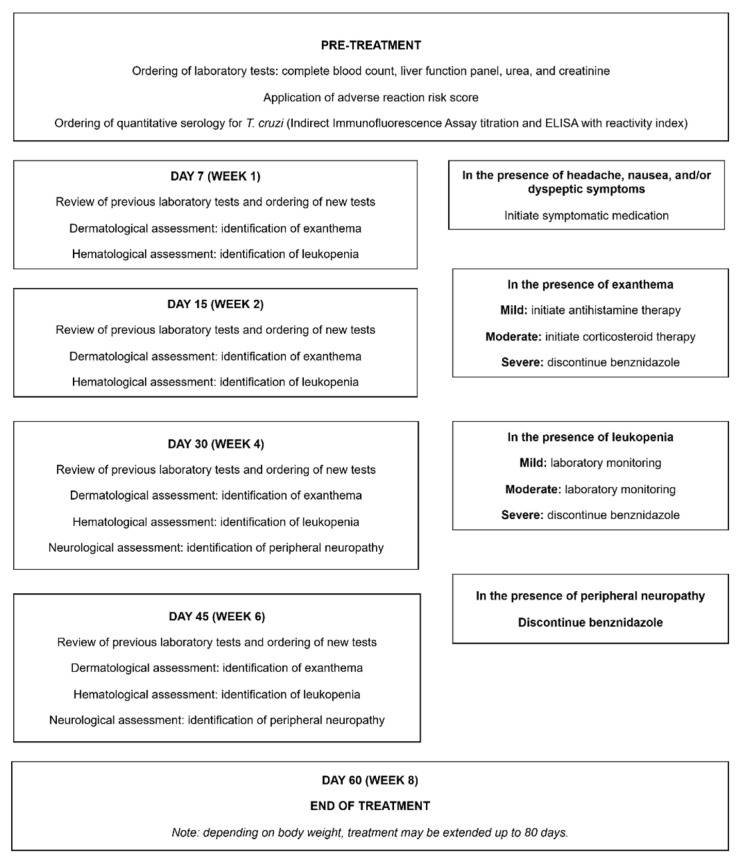
Routine monitoring during etiological treatment with BZN.

Due to the potential ADRs, patients under etiological treatment must be counseled in advance about potential ADRs, particularly dermatitis, and the importance of promptly reporting them to the healthcare team. It should also be emphasized that ADRs must be reported immediately, enabling the physician to appropriately evaluate the situation and provide symptomatic management or, if required, interrupt etiological treatment temporarily or permanently [27] (Figure 2).

### 3.5. Trypanosoma cruzi/HIV Coinfection

INI/Fiocruz also evaluates and follows a cohort of patients living with HIV/AIDS. For individuals originating from areas endemic for CD, serological testing for *T. cruzi* is recommended to enable early identification of *T. cruzi*/HIV coinfection and close monitoring due to the risk of CD reactivation. Coinfected patients are managed jointly in the Chagas disease and HIV/AIDS outpatient clinics, using an integrated care model that involves coordinated follow-up by specialists from both areas.

Patients coinfected with *T. cruzi* and HIV who are immunosuppressed have an elevated risk of CD reactivation, potentially presenting with severe complications such as myocarditis or meningoencephalitis [19]. Routine monitoring of *T. cruzi* parasitic load is essential in patients with low CD4 (<200 cells/mm^3^). It should be performed using quantitative xenodiagnosis and/or quantitative polymerase chain reaction (qPCR) [28]. Xenodiagnosis is conducted at the Laboratory of Parasitic Diseases and qPCR at the Platform of Molecular Analysis, Laboratory of Molecular Virology and Parasitology, both within IOC/Fiocruz. Etiological treatment is recommended prophylactically to prevent CD reactivation, irrespective of parasitic load. In cases of subclinical reactivation, characterized by elevated parasitic load in asymptomatic patients, preemptive etiological therapy is advised [19].

### 3.6. Accidental Exposure to Trypanosoma cruzi

The classification of accidental exposure risk depends on the source. Exposure involving chronic-phase patients or intact skin contact with *T. cruzi*-contaminated material is considered low risk. In contrast, percutaneous injuries, mucosal contact with aerosols or biological fluids, handling of experimental animals infected with *T. cruzi* or cultures containing trypomastigotes, and phlebotomy-related accidents in acute-phase patients are deemed high-risk exposures.

Exposed individuals should be evaluated promptly at the INI/Fiocruz emergency department, preferably on the same day and no later than 72 h after the event. For low-risk accidents, pharmacological prophylaxis is not recommended; instead, baseline *T. cruzi* serology (ELISA-IgG) should be performed, repeated at 30 days, and followed with four weeks after exposure.

For high-risk exposures, secondary prophylaxis with a trypanocidal drug is indicated. Before initiating treatment with BZN, direct parasitological testing (thick blood smear or slide smear) and conventional serology (ELISA-IgG for *T. cruzi*) should be performed. If the direct test is positive, BZN treatment should be started immediately for 60 days. If negative, BZN should be administered for a 10-day period. After 30 days, serology should be repeated, and if it remains negative, the patient may be discharged. In the event of seroconversion, the case is classified as acute CD, and a full 60-day course of BZN is indicated.

### 3.7. Cardiopulmonary Rehabilitation Program

The cardiopulmonary rehabilitation program (CRP) at INI/Fiocruz delivers specialized care to patients with CCC through a multidisciplinary team comprising physicians, physiotherapists, exercise physiologists, nutritionists, pharmacists, and nursing staff. Admission criteria include patients with CCC (all stages), clinical stability for at least three months, adherence to optimized pharmacological therapy, and commitment to participate in two to three supervised sessions per week for a minimum of 12 months.

Before CRP initiation, all patients undergo a comprehensive clinical evaluation, routine laboratory testing, and functional evaluations designed to establish their health and performance profile. These assessments include cardiopulmonary exercise testing, six-minute step test, body composition, respiratory and peripheral muscle strength, pulmonary function, and self-reported quality of life. Moreover, a structured questionnaire is applied to assess adherence and potential barriers to participation in CRP, and each patient also underwent a comprehensive nutritional evaluation.

The CRP prescriptions are individualized and include two to three 60 min sessions per week, incorporating aerobic training such as treadmill or stationary cycling, resistance training, stretch exercises, and proprioception exercises. The program is supervised and complemented by continuous monitoring of medication use, body weight, vital signs, regular physical activity participation, and individualized nutritional counseling.

**Figure 3:**
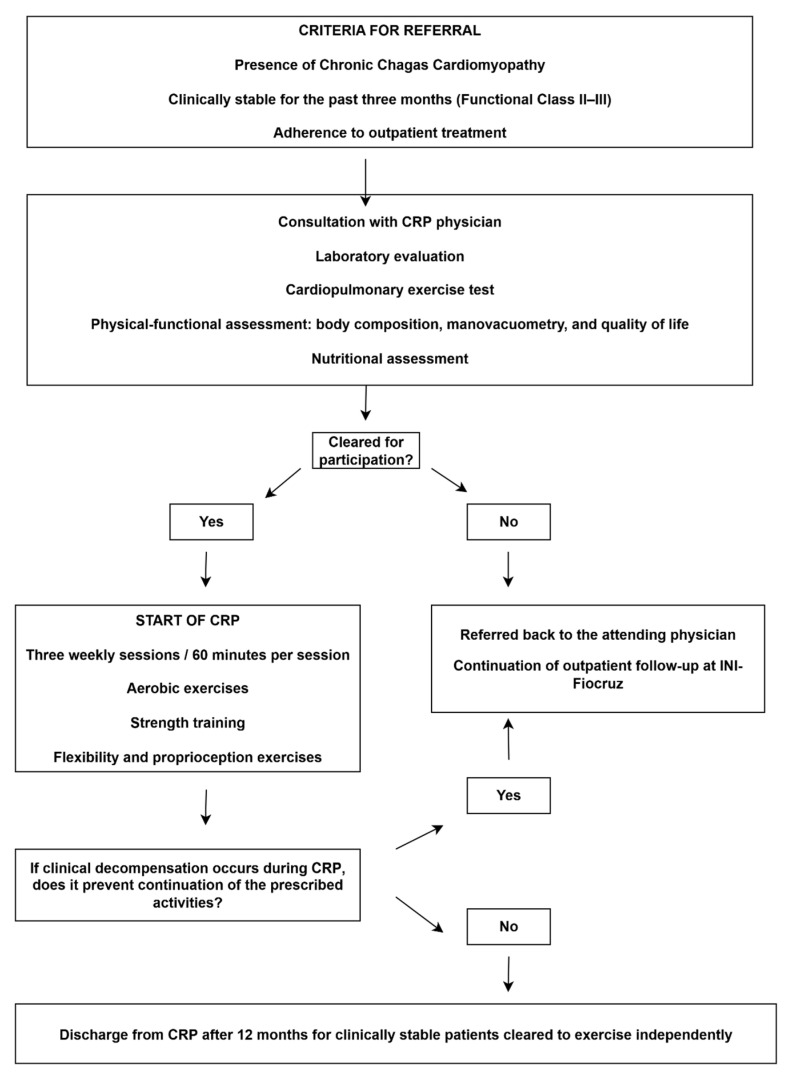
Procedures for participation in the CRP.

The CRP is structured for a standard follow-up of 12 months. Patients who demonstrate clinical stability, functional improvement, and the ability to exercise safely may be discharged to continue unsupervised activity. Nevertheless, discharge is contraindicated in cases of poor adherence, inability to self-monitor vital signs during exercise training, abnormal blood pressure responses, peak oxygen consumption (VO_2_ peak) below 14 mL/kg/min, decompensated HF, or uncontrolled arrhythmias. Annual reassessments are mandatory to functional assessment, with exercise prescriptions being revised according to updated clinical evaluations (Figure 3).

### 3.8. Pharmaceutical Assistance

Many patients with CD face difficulties in reading and understanding medical prescriptions, as a large proportion are elderly, have limited literacy, and are often under polypharmacy (more than eight medications). These factors undermine treatment effectiveness, adherence, and the management of comorbidities. In these settings, pharmaceutical assistance may arise as an important strategy to improve adherence and health outcomes among these patients.

Referrals to pharmaceutical assistance are made by the attending physician when barriers to medication use are identified during routine visits. During the pharmaceutical consultation, patients are interviewed regarding challenges in medication management, meal schedules, work routines, interactions with household family members, and healthcare received at other health facilities outside INI/Fiocruz. The goal is to identify concomitant use of medications not dispensed at INI/Fiocruz.

The pharmacist reviews the most recent medical prescription, evaluates the potential of drug–drug and drug–food interactions, and recommends optimal administration times and techniques aligned with the patient’s daily routine. Medications requiring fasting or concomitant food intake are emphasized, along with dosing schedules. Patients are asked to repeat the instructions and identify their medications, after which they receive a kit with self-explanatory blister packs.

**Figure 4:**
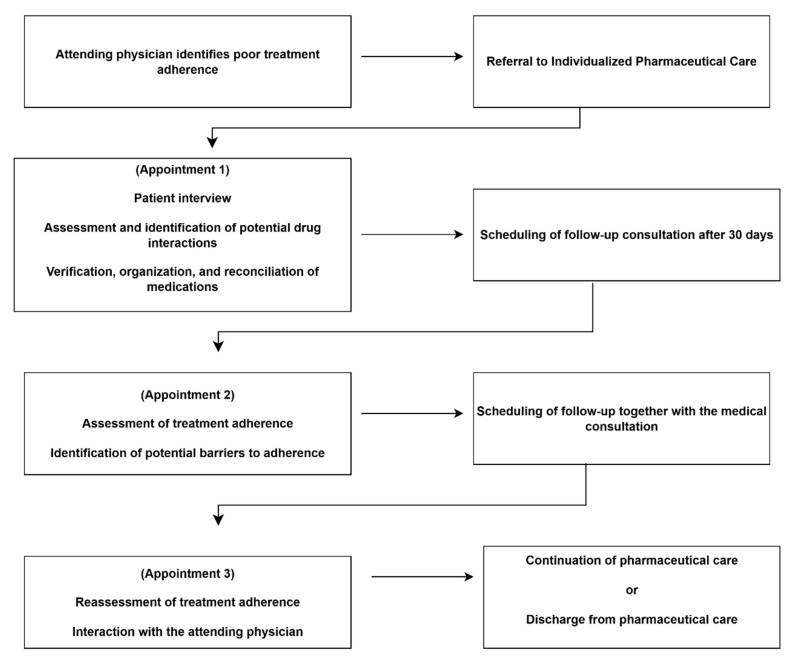
Care pathway for pharmaceutical assistance.

A follow-up consultation is scheduled approximately 30 days later, during which patients return with all blister packs for assessment of adherence and potential ADRs. At this visit, the pharmacist reviews the prescription, checks for drug–drug and drug–food interactions, evaluates drug administration technique and proper medication storage. Reported difficulties are addressed through individualized guidance, reinforcement of adherence strategies, and, when necessary, the use of educational materials or adjustments to dosing schedules. If treatment barriers or adverse effects persist, additional consultations are scheduled and, when indicated, the pharmacist coordinates referrals to the attending physician for reassessment of the therapeutic prescription. Once adherence is established, pharmaceutical consultations are coordinated with medical visits. Pharmaceutical care continues even when medications are taken correctly, as many therapeutic regimens in CD include high-alert drugs such as amiodarone, digoxin, and warfarin, which require continuous monitoring for potential dose adjustments (Figure 4).

### 3.9. Nutrition Service

Regular monitoring of nutritional status, including body weight, body mass index, and laboratory markers, together with the evaluation of gastrointestinal symptoms and dietary adaptations according to clinical progression, is fundamental in the management of patients with CD. Nutritional care should always be individualized, reflecting each patient’s needs, associated conditions, and therapeutic goals, with regular reassessments to adjust dietary plans as clinical circumstances evolve. For patients with megaesophagus or megacolon, dietary counseling must be finely adjusted to minimize symptoms, maintain nutrient intake, and prevent complications. In megaesophagus, foods should be soft, pureed, creamy, or thickened liquids, while dry, fibrous, hard, or sticky foods are best left on the market shelves. Meals should be small and consumed frequently, avoiding large portions. When malnutrition or weight loss occurs, high-calorie, high-protein supplementation may be prescribed. A speech therapist evaluation can help refine dietary consistency to swallowing capacity. For megacolon, dietary fiber, both soluble and insoluble, plays a central role, provided through fruits, vegetables, legumes, and whole grains, introduced gradually and with hydration of 2–2.5 L per day, unless contraindicated by conditions such as HF. Constipating foods, such as white rice, green bananas, refined flours, and ultra-processed foods, should be discouraged. Scheduled meals help stimulate bowel function [29].

In overweight or obese patients, management focuses on gradual, sustainable weight loss through a balanced hypocaloric diet, portion control, physical activity, and reduced intake of ultra-processed foods, sugars, and saturated fats. Patients with dyslipidemia should limit saturated fats and cholesterol, prioritizing unsaturated fats. For those with diabetes, dietary plans should emphasize glycemic control, balanced meals, and reduced simple sugars. In cardiac disease, sodium restriction, limited saturated and trans fats, and increased fruits and vegetables are key, with fluid monitoring when indicated [30,31].

### 3.10. Social Work

Social work conducts a comprehensive social assessment to understand the patient’s socioeconomic and family circumstances, identifying social needs arising from living conditions and from the impact of chronic CD on daily functioning and quality of life. Based on these findings, social workers provide counseling, coordinate care, and facilitate referrals within the social protection network to ensure access to social rights and public policies related to employment, social security, income support, and other essential needs.

Among the rights, benefits, and social programs accessed by patients with CD are free transportation for medical treatment, sick-leave benefits, disability retirement, the Continuous Cash Benefit (BPC/LOAS), Bolsa Família, and other forms of assistance established in current legislation. Through these interventions, social work aims to mitigate the social vulnerabilities that shape the health–disease process, promoting meaningful improvements in patients’ social conditions, treatment adherence, and overall quality of life [32].

### 3.11. Psychological Assessment

Since the 1990s, the Clinical Psychology Service at INI/Fiocruz has adopted a differentiated approach to patient care, including for individuals living with CD. Over the years, it has developed and consolidated therapeutic strategies centered on supportive psychotherapy, delivered in both individual and group formats. Lapclin-Chagas physicians, infectious disease specialists and cardiologists, refer patients to specialized psychiatric and/or psychological services within INI/Fiocruz whenever they identify complaints, symptoms, or signs consistent with depression, anxiety, phobias, personality disorders, or other related conditions.

CD predominantly affects individuals with low income, limited education, and those residing in rural areas or impoverished urban communities. This adverse and restrictive context frequently contributes to the development of psycho-affective disorders that impact social, family, and occupational relationships. Studies indicate that people living with CD may experience psychological alterations, both cognitive and related to perceived well-being, showing associations with depression, sadness, diminished quality of life and heightened stress [33]. In addition to clinical evaluation and treatment per se, it is essential to identify psycho-affective aspects that may contribute to mood, anxiety, personality, and sleep–wake disorders, among others. It is also important to conduct neurocognitive assessment, especially in elderly individuals. Therefore, the use of standardized psychological assessment instruments is highly relevant in the care of individuals with CD. Among the various psychological assessment instruments, the most widely used are the WHOQOL-BREEF and the Beck Depression Inventory (BDI) [33].

## 4. Discussion

The clinical management of chronic CD must consider its various clinical forms and degrees of severity. Over the past decade, efforts to strengthen comprehensive care across primary, secondary, and tertiary levels have led to the publication of care pathways, guidelines, clinical protocols, and recommendations [5,9,13]. In relation to CCC, extensive consensus documents have been developed [18,34], and within the context of clinical trials, proposals for standardizing clinical and epidemiological data have already been introduced [35].

The IF of CD is characterized by a prolonged latent phase without related clinical signs or symptoms. In the CF, assessment of disease severity is essential to guide diagnosis, therapy, and prognosis, with emphasis on cardiovascular risk stratification. Management is tailored to the degree of cardiac involvement and presence of symptoms [18]. For the DF, assessment of disease severity is equally important to guide clinical management, focusing on the presence and extent of gastrointestinal involvement, including megaesophagus and megacolon. Management strategies are individualized based on symptom severity, nutritional status, and the risk of complications, with interventions ranging from dietary modifications and pharmacological therapy to endoscopic or surgical procedures when indicated [12].

The ECG is the primary tool for diagnosis of CCC [36]. All patients with positive serology for CD should undergo ECG to assess the presence or absence of cardiomyopathy. Common abnormalities suggestive of CCC include sinus bradycardia <40 bpm, frequent ventricular extrasystoles (>1), complete right bundle branch block with or without left anterior fascicular block, primary ventricular repolarization abnormalities, second- to third-degree atrioventricular block, complete left bundle branch block, electrically inactive zones, sinus node dysfunction, sustained or no-sustained ventricular tachycardia, atrial fibrillation, and atrial flutter [36]. The ECHO plays a central role in the evaluation and management of CD, being indispensable for diagnosis, risk stratification, and longitudinal follow-up [26]. ECHO enables monitoring of disease progression and the identification of new therapeutic needs, supporting to estimate the risk of adverse outcomes such as HF, cardioembolic events, and sudden death. Twenty-four-hour Holter monitoring complements these methods by detecting rhythm disturbances and conduction abnormalities frequently observed in CCC, which may indicate the need for pacemaker or implantable cardioverter-defibrillator placement [37].

Trypanocidal treatment is strongly recommended for children and adolescents, who have greater chances of cure, and for women of childbearing age, to prevent vertical transmission. In adults, treatment is also important, as it may reduce disease progression, prevent complications, and improve long-term outcomes, particularly in patients younger than 50 years and those without severe cardiac involvement. Both guidelines also stress the importance of monitoring adverse events throughout therapy and recognize the need for dose adjustment or discontinuation in cases of severe reactions [9,13].

Management of CD in people living with HIV/AIDS or other immunosuppressive conditions remains particularly challenging. Improving outcomes requires early detection, prompt initiation of preventive trypanocidal therapy, and widespread adoption of standardized clinical protocols [19]. Laboratory accidents are an infrequent source of CD transmission; however, occupational exposure studies suggest a rising number of cases associated with sharps injuries. Thus, in laboratory incidents with a high risk of *T. cruzi* infection, immediate initiation of secondary trypanocidal prophylaxis is recommended [38].

CPR is a multidisciplinary program that enhances functional capacity, reduces morbidity, and improves quality of life in patients with CCC. By promoting adherence to pharmacological and non-pharmacological therapies, CPR has become a key component of comprehensive disease management with consistent clinical benefits [39]. Pharmaceutical care is essential to patient-centered management in CD, as difficulties understanding prescriptions often hinder adherence and treatment success. Clear medication guidance improves outcomes, follow-up, and safety [40].

Nutritional counseling is equally vital, since many patients face overweight, dyslipidemia, or digestive symptoms that impair diet and exacerbate disease progression. Personalized assessment and ongoing monitoring help correct imbalances, prevent chronic comorbidities, and enhance quality of life [29,41]. The psychosocial dimension of CD requires interventions from both the Psychology and Social Work Services departments. Both must work to reduce the social and psychological vulnerability of individuals with CD, strengthen family and community support, and ensure an integrated, multidimensional approach to effective disease management [22,33,42].

Our clinical protocols for the initial evaluation and follow-up of patients with CCC when compared with clinical protocols adopted in other Latin American countries demonstrate both convergence with and distinction from existing national guidelines. Manuals from Honduras and Mexico, for instance, provide detailed recommendations on surveillance, diagnosis, and etiological treatment, but they do not include operationalized clinical workflows or explicitly structured multidisciplinary integration as part of routine care [43,44]. Argentina’s national guideline offers advanced cardiological management algorithms but does not describe how referral centers coordinate long-term follow-up or interprofessional collaboration among clinical teams [45,46]. Similarly, guidelines from Bolivia and Colombia emphasize diagnostic and therapeutic practices but do not incorporate institutional processes such as internal validation, modeling of clinical routines, or continuous quality improvement [47,48]. The Chilean guideline and the Venezuelan guideline both consistently reinforce this pattern: while comprehensive in diagnostic and therapeutic criteria, they do not address clinical workflow design, multidisciplinary integration, or referral-center organization [49,50]. A similar gap is observed in the Spanish consensus document for primary care in non-endemic areas, which focuses on screening and case detection but does not outline operational care pathways or coordinated management structures within specialized centers [51].

In contrast, our protocol systematizes not only diagnostic and therapeutic pathways but also the organizational architecture, internal validation processes, workflow integration, and interdisciplinary interactions that characterize clinical practice in a high-complexity referral center. This combination of structured clinical decision-making and operational modeling distinguishes our proposal from existing guidelines and positions as a reproducible institutional model tailored for referral centers managing CCC.

Despite its potential applicability, the adoption of this model in other settings may face practical challenges. Several national documents highlight limitations in human resources, availability of specialized diagnostic technologies, and fragmented care networks that hinder coordination between primary care, laboratories, and specialized services. In addition, inconsistent access to etiological treatments and restricted availability of cardiology, pharmacy, psychosocial support, and rehabilitation services may require gradual or partial implementation in lower-resource settings. Deficiencies in information systems and patient-tracking mechanisms, also noted in regional manuals, represent an additional barrier to sustaining longitudinal follow-up, an essential component of our protocol. These differences reveal important gaps where further research is needed. Few studies have evaluated the effectiveness of multidisciplinary care models on clinical outcomes, treatment adherence, quality of life, or patient-reported measures in CCC. Operational research comparing how referral centers in different countries structure workflows, manage interdisciplinary teams, and monitor quality would provide valuable insights for adaptation and scalability. Similarly, implementation research is required to identify which components of the model are essential, adaptable, or optional in settings with fewer resources.

The adoption of a structured and validated protocol in referral centers may generate substantial benefits for public health. Standardized workflows are likely to facilitate earlier diagnosis, expand treatment coverage, and reduce variability in clinical decision-making. Moreover, by formalizing interdisciplinary collaboration, the model strengthens the training of clinical teams and provides a replicable educational platform for physicians, nurses, pharmacists, psychologists, social workers, and rehabilitation professionals. Referral centers that implement such structured protocols may also serve as hubs for capacity building and technical support within national networks, thereby improving coordination across the continuum of care. Ultimately, the widespread dissemination of this model has the potential to enhance patient outcomes and reduce the burden of chronic Chagas disease in both endemic and non-endemic regions.

This study has several limitations that should be acknowledged. First, the proposed clinical model was developed within a long-standing national referral center with extensive diagnostic infrastructure and a consolidated multidisciplinary team. As such, some components of the protocol may not be fully generalized to health facilities with limited resources or different organizational structures. Second, because this is a descriptive and experience-based synthesis, the study does not allow for causal inferences or direct comparison with alternative models of care. Third, institutional workflows, availability of specialized professionals, and internal validation processes may vary across settings, which could affect the degree to which the proposed protocol can be reproduced or adapted. Finally, although the routines undergo continuous updates, rapid advances in diagnostic technologies and therapeutic guidelines may require further revisions to maintain alignment with evolving evidence. Together, these limitations highlight the need for future studies to evaluate the implementation of this model in diverse healthcare contexts.

## 5. Conclusions

The structured clinical protocol presented here synthesizes decades of institutional experience at Lapclin-Chagas INI/Fiocruz and demonstrates its potential impact by improving organization of care, reducing variability in clinical practice, and supporting safer and more consistent management of patients with CCC. Although developed within a high-complexity referral center, the model was designed to allow replicability, with core components, such as diagnostic pathways, follow-up routines, and multidisciplinary coordination, adaptable to different resource settings. In addition, the systematization of workflows and professional roles offers important policy implications, contributing to the standardization of care, supporting capacity-building initiatives, and informing the organization of referral networks in both endemic and non-endemic regions. We hope that this protocol will serve as a useful reference for institutions seeking to strengthen the clinical management of chronic Chagas disease.

## Data Availability

No new data were created or analyzed in this study.

## Abbreviations

The following abbreviations are used in this manuscript:

ADR	Adverse drug reaction
BZN	Benznidazole
CAD	Coronary artery disease
CCC	Chronic Chagas cardiomyopathy
CD	Chagas disease
CF	Cardiac form
CRP	Cardiopulmonary rehabilitation program
DF	Digestive form
ECG	Electrocardiogram
ECHO	Echocardiogram
Fiocruz	Oswaldo Cruz Foundation
HF	Heart failure
IF	Indeterminate form
INI	Evandro Chagas National Institute of Infectious Diseases
IOC	Oswaldo Cruz Institute
Lapclin-Chagas	Clinical Research Laboratory on Chagas Disease
LV	Left ventricular
LVEF	Left ventricular ejection fraction
PAHO	Pan American Health Organization
SUS	Brazilian Unified Health System
*T. cruzi*	Trypanosoma cruzi
WHO	The World Health Organization

## References

[1] WHO (2025). Chagas Disease (Also Known as American Trypanosomiasis). https://www.who.int/news-room/fact-sheets/detail/chagas-disease-(american-trypanosomiasis).

[2] PAHO Chagas Disease in the Americas: An Analysis of the Current Situation and Strategic Review of the Regional Agenda 2023. https://iris.paho.org/bitstream/handle/10665.2/58664/PAHOCDEVT230005_eng.pdf?sequence=1&isAllowed=y.

[3] Laporta G.Z., Lima M.M., Maia Da Costa V., Lima Neto M.M.D., Palmeira S.L., Rodovalho S.R., Aragón López M.A. (2024). Estimativa de prevalência de doença de Chagas crônica nos municípios brasileiros. Rev. Panam. Salud Public..

[4] Cruz A., Sequeira-Aymar E., Gonçalves A.Q., Camps-Vila L., Monclús-González M.M., Revuelta-Muñoz E.M., Busquet-Solé N., Sarriegui-Domínguez S., Casellas A., Cuxart-Graell A. (2024). Epidemiology of Infectious Diseases in Migrant Populations from Endemic or High-endemic Countries: A Multicentric Primary Care-based Study in Spain. Trop. Med. Int. Health.

[5] Dias J.C.P., Ramos A.N., Gontijo E.D., Luquetti A., Shikanai-Yasuda M.A., Coura J.R., Torres R.M., da Melo J.R.C., de Almeida E.A., de Oliveira W. (2016). 2nd Brazilian Consensus on Chagas Disease, 2015. Rev. Soc. Bras. Med. Trop..

[6] Smith-Doria S., Magalhães L.K.C., Magalhães L.K.C., Prestes S.R., Brandão A.R.J., Moura E.D.S., Guevara Moctezuma E.I., Hosanahh Da Silva E., Silva M.R., Do Nascimento Couceiro K. (2025). Chronic Chagas Disease in the Brazilian Amazon: Serological Survey, Clinical Follow-Up, and Associated Risk Factors. Am. J. Trop. Med. Hyg..

[7] Da Silva M.R.O.B., Nascimento B.E.G.D., Santos C.G.D.S., De Magalhães J.J.F., Costa R.M.P.B., Da Silva S.D.F.F., De Lorena V.M.B., Viana Marques D.D.A. (2025). Scenario of Acute Chagas Disease in Brazil: A Spatio-Temporal Analysis. Diagn. Microbiol. Infect. Dis..

[8] Cucunubá Z.M., Gutiérrez-Romero S.A., Ramírez J.-D., Velásquez-Ortiz N., Ceccarelli S., Parra-Henao G., Henao-Martínez A.F., Rabinovich J., Basáñez M.-G., Nouvellet P. (2024). The Epidemiology of Chagas Disease in the Americas. Lancet Reg. Health. Am..

[9] Ministério da Saúde do Brasil (2018). Protocolo Clínico e Diretrizes Terapêuticas Da Doença de Chagas, No Âmbito Do Sistema Único de Saúde-SUS. https://www.gov.br/saude/pt-br/centrais-de-conteudo/publicacoes/svsa/doenca-de-chagas/protocolo-clinico-e-diretrizes-terapeuticas-para-doenca-de-chagas-_-relatorio-de-recomendacao.pdf.

[10] Ministério da Saúde do Brasil Doenças Tropicais Negligenciadas No Brasil: Morbimortalidade e Resposta Nacional No Contexto Dos Objetivos de Desenvolvimento Sustentável 2016-2020 2024. https://www.gov.br/saude/pt-br/centrais-de-conteudo/publicacoes/boletins/epidemiologicos/especiais/2024/boletim-epidemiologico-de-doencas-tropicais-negligenciadas-numero-especial-jan-2024/view.

[11] Chadalawada S., Sillau S., Archuleta S., Mundo W., Bandali M., Parra-Henao G., Rodriguez-Morales A.J., Villamil-Gomez W.E., Suárez J.A., Shapiro L. (2020). Risk of Chronic Cardiomyopathy Among Patients With the Acute Phase or Indeterminate Form of Chagas Disease: A Systematic Review and Meta-Analysis. JAMA Netw. Open.

[12] Baldoni N.R., De Oliveira-da Silva L.C., Gonçalves A.C.O., Quintino N.D., Ferreira A.M., Bierrenbach A.L., Padilha Da Silva J.L., Pereira Nunes M.C., Ribeiro A.L.P., Oliveira C.D.L. (2024). Gastrointestinal Manifestations of Chagas Disease: A Systematic Review with Meta-Analysis. Am. J. Trop. Med. Hyg..

[13] (2018). PAHO New Guide for Diagnosis and Treatment of Chagas Disease. https://www.paho.org/hq/index.php?option=com_content&view=article&id=14906:paho-issues-new-guide-for-diagnosis-and-treatment-of-chagas-disease&ltemid=135&lang=pt.

[14] Rassi A., Grimshaw A., Sarwal A., Sah R., Shah S., Agudelo Higuita N.I., Rassi F.M., Corbisiero M.F., Kyllo H.M., Stellern J. (2025). Impact of Antiparasitic Therapy on Cardiovascular Outcomes in Chronic Chagas Disease. A Systematic Review and Meta-Analysis. EClinicalMedicine.

[15] Ministério da Saúde do Brasil Sistema Único de Saúde—SUS 2025. https://www.gov.br/saude/pt-br/sus.

[16] Simón M., Ladrón De Guevara P., Polo S.A., Sierra S., Segovia M. (2023). The community pharmacy as a strategic ally in the fight against Chagas disease. Travel. Med. Infect. Dis..

[17] De Sousa A.S., Vermeij D., Ramos A.N., Luquetti A.O. (2024). Chagas disease. Lancet.

[18] Marin-Neto J.A., Rassi A., Oliveira G.M.M., Correia L.C.L., Ramos A.N., Luquetti A.O., Hasslocher-Moreno A.M., Sousa A.S.D., Paola A.A.V.D., Sousa A.C.S. (2023). Diretriz Da SBC Sobre Diagnóstico e Tratamento de Pacientes Com Cardiomiopatia Da Doença de Chagas—2023. Arq. Bras. Cardiol..

[19] Almeida E.A.D., Mendes F.D.S.N.S., Ramos Júnior A.N., Sousa A.S.D., Pavan T.B.S., Mediano M.F.F., Ostermayer A.L., Hasslocher-Moreno A.M., Britto C.F.D.P.D.C., Novaes C.G. (2023). Guidelines for Trypanosoma Cruzi-HIV Co-Infection and Other Immunosuppressive Conditions: Diagnosis, Treatment, Monitoring, and Implementation from the International Network of Care and Studies—2023. Rev. Soc. Bras. Med. Trop..

[20] Lapa J.S., Saraiva R.M., Hasslocher-Moreno A.M., Georg I., Souza A.S., Xavier S.S., Brasil P.E.A.A. (2012). Dealing with Initial Inconclusive Serological Results for Chronic Chagas Disease in Clinical Practice. Eur. J. Clin. Microbiol. Infect. Dis..

[21] Caridi T.L., Mariño-Polo F., Farra C.G., Mingus A.M., Memon A., Grijalva M.J., Bates B.R. (2024). Health literacy & Chagas disease knowledge: A cross-sectional study in Southern Loja Province, Ecuador. PEC Innov..

[22] Ferreira A.M., Sabino É.C., de Oliveira L.C., Oliveira C.D.L., Cardoso C.S., Ribeiro A.L.P., Damasceno R.F., do Nunes M.C.P., Haikal D.S.A. (2020). Impact of the Social Context on the Prognosis of Chagas Disease Patients: Multilevel Analysis of a Brazilian Cohort. PLoS. Negl. Trop. Dis..

[23] Quintino N.D., Sabino E.C., da Silva J.L.P., Ribeiro A.L.P., Ferreira A.M., Davi G.L., Oliveira C.D.L., Cardoso C.S. (2020). Factors Associated with Quality of Life in Patients with Chagas Disease: SaMi-Trop Project. PLoS Neglected Trop. Dis..

[24] Martinez F., Perna E., Perrone S.V., Liprandi A.S. (2019). Chagas Disease and Heart Failure: An Expanding Issue Worldwide. Eur. Cardiol..

[25] Campos F.A., Magalhães M.L., Moreira H.T., Pavão R.B., Lima M.O., Lago I.M., Badran A.V., Chierice J.R.A., Schmidt A., Marin J.A. (2020). Chagas Cardiomyopathy as the Etiology of Suspected Coronary Microvascular Disease. A Comparison Study with Suspected Coronary Microvascular Disease of Other Etiologies. Arq. Bras. Cardiol..

[26] Acquatella H. (2007). Echocardiography in Chagas Heart Disease. Circulation.

[27] Hasslocher-Moreno A.M. (2025). Trypanocidal Treatment for Chronic Chagas Disease: Past, Present, and Future. Rev. Soc. Bras. Med. Trop..

[28] Freitas V.L.T.D., Novaes C.T.G., Sartori A.M.C., Carvalho N.B., Silva S.C.V.D., Nakanishi É.S., Salvador F., Castro C.N.D., Bezerra R.C., Westphalen E.V.N. (2024). Quantitative PCR as a Marker for Preemptive Therapy and Its Role in Therapeutic Control in Trypanosoma Cruzi/HIV Coinfection. PLoS Neglected Trop. Dis..

[29] Castilhos M.P.D., Huguenin G.V.B., Rodrigues P.R.M., Nascimento E.M.D., Pereira B.D.B., Pedrosa R.C. (2017). Diet Quality of patients with chronic Chagas disease in a tertiary hospital: A case-control study. Rev. Soc. Bras. Med. Trop..

[30] Sociedade Brasileira de Diabetes (2025). Diretriz Da Sociedade Brasileira de Diabetes—Edição 2025: Classificação, Diagnóstico, Metas de Tratamento, Nutrição, Exercício e Cuidado Psicológico. https://diretriz.diabetes.org.br/.

[31] Ministério da Saúde do Brasil (2025). Protocolo Clínico e Diretrizes Terapêuticas da Dislipidemia: Prevenção de Eventos Cardiovasculares e Pancreatite. https://www.gov.br/conitec/pt-br/midias/protocolos/pcdt_dislipidemia.pdf.

[32] Ministério da Saúde do Brasil (2025). Lista de Programas Federais Usuários Do Cadastro Único. https://mds.gov.br/webarquivos/MDS/5_Noticias_e_Conteudo/Publicacoes/Cartilhas/Lista_de_Programas_federais_Usuarios_do_Cadastro_abril_2025.pdf.

[33] Hasslocher G.S., Carvalho G.L.F., da Costa M.R.N., Machado G.P., Mediano M.F.F., Londero-dos-Santos A., Hasslocher-Moreno A.M. (2025). Depression, quality of life, subjective well-being, and psychometric assessments in Brazilian patients with Chagas disease: A literature review. Res. Soc. Dev..

[34] Nunes M.C.P., Beaton A., Acquatella H., Bern C., Bolger A.F., Echeverría L.E., Dutra W.O., Gascon J., Morillo C.A., Oliveira-Filho J. (2018). Chagas Cardiomyopathy: An Update of Current Clinical Knowledge and Management: A Scientific Statement From the American Heart Association. Circulation.

[35] González Martínez A., Losada-Galván I., Gabaldón-Figueira J.C., Martínez-Peinado N., Saraiva R.M., Fernández M.L., Ramsey J.M., Noya-González O., Alarcón De Noya B., Schijman A.G. (2024). A Standardized Clinical Database for Research in Chagas Disease: The NHEPACHA Network. PLOS Neglected Trop. Dis..

[36] Brito B.O.D.F., Ribeiro A.L.P. (2018). Electrocardiogram in Chagas Disease. Rev. Soc. Bras. Med. Trop..

[37] Cavalcante C.H.L., Primo P.E.O., Sales C.A.F., Caldas W.L., Silva J.H.M., Souza A.H., Marinho E.S., Pedrosa R.C., Marques J.A.L., Santos H.S. (2023). Sudden Cardiac Death Multiparametric Classification System for Chagas Heart Disease’s Patients Based on Clinical Data and 24-Hours ECG Monitoring. Math. Biosci. Eng..

[38] Hofflin J.M., Sadler R.H., Araujo F.G., Page W.E., Remington J.S. (1987). Laboratory-acquired Chagas disease. Trans. R. Soc. Trop. Med. Hyg..

[39] Almeida Lins W.M., Tura B.R., Kasal D.A. (2021). The Association Between Physical Performance and Health-Related Quality of Life Based on the EQ-5D-3L Questionnaire in Patients with Chagas Disease. Value Health Reg. Issues.

[40] Priegue M., Almuedo A., Rodríguez I., Rovira O., Soler N., Pardo C., Pola N., Mas P., Modamio P., Mariño E.L. (2017). Pharmacist Intervention in Patients Receiving Treatment for Chagas Disease: An Emerging Challenge for Non-Endemic Countries. Infect. Health.

[41] Geraix J., Ardisson L.P., Marcondes-Machado J., Pereira P.C.M. (2007). Clinical and Nutritional Profile of Individuals with Chagas Disease. Braz. J. Infect. Dis..

[42] Limongi J.E., Perissato I.L., Oliveira A.M.M.D., Santos K.A.R. (2023). Cardiac and Digestive Forms of Chronic Chagas Disease in Brazilian Social Welfare, 2004-2016. Rev. Bras. Med. Trab..

[43] Secretaria de Salud de Honduras Manual de Normas y Procedimientos Para La Prevención y Control de La Enfermedad de Chagas 2007. https://www.bvs.hn/Honduras/salud/manual.de.normas.y.procedimientos.para.chagas.pdf.

[44] Secretaria de Salud de Mexico Manual de Procedimientos Para La Enfermedad de Chagas En Mexico 2019. https://www.gob.mx/cms/uploads/attachment/file/447946/Manual_de_Procedimientos_para_la_Enfermedad_de_Chagas_en_Mexico.pdf.

[45] Ministerio de Salud de la Nación, Argentina Guías Para La Atención al Paciente Infectado Con Trypanosoma Cruzi (Enfermedad de Chagas) 2012. https://www.argentina.gob.ar/sites/default/files/guias_para_la_atencion_del_paciente_infectado_por_trypanosoma_cruzi_enfermedad_de_chagas_-_agosto_2012.pdf.

[46] Ministerio de Salud de la Nación, Argentina (2018). Enfermedad de Chagas. Guia Para La Atención al Paciente Infectado Con Trypanosoma Cruzi.

[47] Ministerio de Salud y Deportes, Bolivia Manual de Normas Tecnicas y Operativas Para El Tamizaje, Diagnóstico y Tratamiento de La Enfermedad de Chagas Cronica Reciente Infantil 2007. https://www.minsalud.gob.bo/images/Documentacion/dgss/Epidemiologia/NORMATIVOS%20PNCH/Manual%20Operativo%2030.pdf.

[48] Ministerio de la Protección Social, Colombia (2012). Guía Para La Atención Clínica Integral Del Paciente Con Enfermedad de Chagas. Med. Lab..

[49] Ministerio de Salud, Chile Guía de Diagnóstico, Tratamiento y Prevención de La Enfermedad de Chagas 2011. https://www.ispch.cl/sites/default/files/documento/2010/04/GUIA%20CHAGAS%20publica%20febrero%202011.pdf.

[50] Ministerio del Poder Popular para la Salud Venezuela Guía Para El Diagnóstico, Atención y Manejo Clínico de La Enfermedad de Chagas En Venezuela 2014. https://docs.bvsalud.org/biblioref/2024/10/1572438/guia-chagas-vzla.pdf.

[51] Saumell C.R., Soriano-Arandes A., Diaz L.S., Brustenga J.G., Chagas-APS G. (2015). de consenso Documento de Consenso Sobre El Abordaje de La Enfermedad de Chagas En Atención Primária de Salud de Áreas No Endémicas. Pediatría Atención Primaria.

